# Nutritional diagnoses in people with type 2 diabetes: association with metabolic, anthropometric, and dietary parameters

**DOI:** 10.3389/fnut.2024.1473429

**Published:** 2024-10-18

**Authors:** María Victoria Landa-Anell, Fabiola M. Del Razo-Olvera, Irene Bodnar, Brenda Cordova-Isidro, Daniela Lagunas-Valdepeña, Ana T. Arias-Marroquín, Ana Cristina García-Ulloa, Marco Antonio Melgarejo-Hernández, Sergio Hernández-Jiménez, for the Group of Study CAIPaDi

**Affiliations:** ^1^Centro de Atención Integral del Paciente con Diabetes (CAIPaDi) del Instituto Nacional de Ciencias Médicas y Nutrición Salvador Zubirán, Mexico City, Mexico; ^2^Unidad de investigación de Enfermedades Metabólicas del Instituto Nacional de Ciencias Médicas y Nutrición Salvador Zubirán, Mexico City, Mexico

**Keywords:** nutritional diagnoses, type 2 diabetes, nutritional care process, terminology of the nutritional care process, medical nutrition therapy, biochemical markers, anthropometry

## Abstract

**Background:**

Nutritional diagnosis involves identifying a nutritional problem, its cause, and the signs that indicate it to guide appropriate treatment. Few studies report on the most prevalent nutritional diagnoses in people living with type 2 diabetes (T2D).

**Objective:**

To define nutritional diagnoses across different domains and their association with metabolic, anthropometric, and dietary parameters in individuals with T2D.

**Methods:**

A personalized nutritional intervention was conducted using the Nutrition Care Process (NCP) model, which encompasses assessment, diagnosis, intervention, and evaluation, utilizing standardized terminology from the Nutrition Care Process Terminology (NCPT). Two dietitians, trained and standardized in applying the NCP and NCPT, performed patient assessments and established the diagnoses. Patients over 18 years old with a diagnosis of T2D for less than 5 years were included.

**Results:**

Data from 2,050 patients were analyzed, of whom 55.3% were women, and 44.7% were men, with a median age of 57 and 54 years, respectively. The most prevalent nutritional diagnosis was excessive energy and carbohydrate intake. Diagnoses were distributed across domains: Intake (55.9%), Behavioral/Environmental (32.7%), Clinical (10.2%), and 1.2% without nutritional diagnosis. Significant intergroup differences were observed in anthropometric variables such as BMI, waist circumference, and body fat percentage (*p* < 0.05). HbA1c and glucose levels were significantly higher in the Intake and Behavioral groups (*p* < 0.001). Albumin/creatinine ratio (ACR) was higher in the ingestion group (*p* = 0.007). Caloric and carbohydrate intake were higher in the ingestion group, while protein and fat intake were higher in the clinical and behavioral groups (*p* < 0.001).

**Conclusion:**

Nutritional diagnoses in the intake domain, followed by behavioral/ environmental and clinical domains, are highly prevalent in people with T2D and are associated with worse metabolic control, higher BMI, and increased energy and carbohydrate intake. Timely identification of issues in these domains can support targeted nutritional therapy to improve disease management and promote a healthy lifestyle.

**Clinical trial registration:**

https://clinicaltrials.gov/study/NCT02836808?term=caipadi&rank=2, Identifier (NCT02836808).

## Introduction

Diabetes is one of the most prevalent diseases worldwide, with 14.6 million individuals currently affected in México. According to the 2021 Global Burden of Disease (GBD) report, diabetes is a leading cause of health loss in Mexico, posing a significant challenge to the healthcare system (1).

Medical nutrition therapy (MNT) is a cornerstone in preventing and managing T2D. MNT involves applying evidence-based strategies to address specific nutritional problems at individual and population levels. The Nutrition Care Process (NCP) consists of four main steps: Nutrition Assessment, Nutrition Diagnosis, Nutrition Intervention, and Nutrition Monitoring and Evaluation, and the Nutrition Care Process Terminology (NCPT), which provides standardized terms for each of these steps are key tools for implementing MNT in clinical settings, facilitating the identification, documentation, and resolution of nutritional problems (2). These tools standardize the assessment and monitoring of patient outcomes, ensuring personalized and high-quality nutritional care (3).

In recent years, research documenting the implementation of NCP in nutrition interventions has shown promising results. It highlights its potential to improve the quality of care through a structured and standardized approach, allowing for easier tracking of outcomes (4, 5).

At the CAIPaDI (Centro de Atención Integral del Paciente con Diabetes), the Nutrition Service has integrated the NCP into its intervention model. This approach allows for patient-centered assessments, prioritizing nutritional diagnoses according to NCPT across its domains and subdomains (6). Nutritional assessment enables the nutritionist to develop precise nutritional diagnoses (7). A nutrition diagnosis identifies a nutrition-related problem based on a comprehensive evaluation of the individual’s nutritional status. This includes determining the specific issue, identifying the underlying etiology, and describing the observable signs and symptoms that evidence the problem (8). For example, *a nutritional diagnosis: Inadequate energy intake relates to only drinking liquids due to swallowing difficulties and nausea, evidenced by a patient's energy intake being lower than 70% of the estimated need*.

Several studies have demonstrated the effects of personalized nutritional interventions on glycemic control in patients with T2D, showing that such approaches are more effective in improving parameters like HbA1c and fasting glucose (9). However, no studies have specifically evaluated the relationship between nutritional diagnoses and key factors such as metabolic control, body composition, or dietary intake in the Mexican population (10). Therefore, this study aims to describe the most common nutritional diagnoses and their relationship with metabolic, anthropometric, and dietary parameters in individuals living with T2D receiving care through an integrated patient management program.

## Methods

This exploratory study aimed to assess the frequency of nutritional diagnoses across different domains of the NCP and their correlation with anthropometric, biochemical, and dietary parameters among participants attending the CAIPaDi at the Instituto Nacional de Ciencias Médicas y Nutrición Salvador Zubirán (INCMNSZ). The CAIPaDi program is designed to achieve metabolic goals and provide education and empowerment techniques over a short period, followed by remote support through email or mobile phone technology. Participants in this program receive care from multiple specialties, including nutrition services. The target population consists of patients over 18 years old with type 2 diabetes, free of chronic complications, including those with a diagnosis of T2D for less than 5 years, with a BMI under 45 kg/m^2^, and non-smokers (10).

Data collection was collected from February 2016 to September 2019. Informed consent was obtained from all participants, using good clinical practices and local regulations, before initiating any study procedures. Each participant was assigned a unique patient ID to maintain the confidentiality of personal data. The CAIPaDi program was approved by the institutional Ethics and Research Committees of the INCMNSZ (Ref., -1198), with registration in ClinicalTrials.gov (NCT02836808) (10, 11).

In the nutrition service, personalized nutritional interventions are conducted for all participants according to the NCP model framework (assessment, diagnosis, intervention, and evaluation), utilizing the standardized Nutrition Care Process Terminology (NCPT). The intervention was implemented by two dietitians, who were standardized in applying the NCP and proficient in using the associated terminology.

### Population and sample

The study included 2,050 patients who attended their baseline visit of the CAIPaDi program. Hernández et al. previously published the program’s complete characteristics (11).

Patients fasted for 9 to 12 h before blood sample collection. Measurements included glucose, lipid profile (triglycerides, total cholesterol, LDL cholesterol, HDL cholesterol), non-HDL cholesterol (total cholesterol–HDL cholesterol), albumin/creatinine ratio (ACR; SYNCHRON CX system with colorimetric technique), and glycated hemoglobin (HbA1c; Bio-Rad Variant II Turbo HbA1c Kit 2, with HPLC technique). The central laboratory is certified by ISO 9001:2015 and the College of American Pathologists. Bioelectrical Impendence Analysis (BIA) was used to estimate body composition, specifically weight, body fat percentage, and lean mass. The results were interpreted according to standardized protocols, considering variables such as age, sex, and BMI to ensure accuracy. The JAWON Medical IOI353 body composition analyzer was utilized.

### Application of the nutrition care process model

Data collection for the evaluation of the subjects was conducted according to the Nutrition Care Process (NCP) model and the Nutrition Care Process Terminology (NCPT) to standardize the terminology used for nutritional diagnoses. Nutritional assessment was performed after analyzing anthropometric measurements (weight, height, waist, and hip circumference) and body composition (percentage of body fat, lean mass, fat-free mass) as well as biochemical and clinical data (previous diagnoses and medications use).

Dietary information was obtained through a self-administered three-day food record (two weekdays and one weekend) before the visit. This tool allowed a quantitative analysis (average kilocalories consumed and their macronutrient distribution) and a qualitative analysis of their diet (food choices). Additionally, a questionnaire on barriers to adhering to the dietary plan was administered, which helped to identify key points that may difficult compliance. Understanding these barriers helps prioritize the nutritional problem and make a diagnosis considering various aspects of their lifestyle (12).

### Nutritional diagnosis

The nutritional diagnosis was conducted using the PES format (Problem, Etiology/Cause, and Signs and symptoms), which consists of three components: (1) P: Identify the problem, which describes an alteration in the patient’s condition and is accompanied by a descriptor such as “altered,” “excessive” or “inadequate” (2) E: Determine etiology/cause, which identifies the factors contributing to the existence of the alteration and is linked to the problem label using the words “related to”; and (3) S: State of signs & symptoms, which are the defining characteristics that provide evidence of the existence of the problem (13).

### Nutritional diagnosis classification using the NCPT

The terminology of the Nutrition Care Process Terminology (NCPT) classifies nutritional problems into three fundamental dimensions: (a) Ingestion domain, which is related to current issues regarding the intake of energy, nutrients, liquids, or bioactive substances obtained through an oral diet or nutritional support; (b) Clinical domain, which refers to nutritional problems or findings identified that are related to specific medical or physical conditions; and (c) Behavioral or Environmental domain, which are associated with nutritional problems or findings related to knowledge, attitudes/beliefs, the physical environment, food access, or food security (13). The NCPT also allows us to classify a patient as having no apparent nutritional problem during the evaluation, meaning that the client may be considered without a nutritional diagnosis until a subsequent evaluation (13).

### Statistical analysis

The data distribution was assessed using the Kolmogorov–Smirnov normality test. Participant characteristics were detailed using medians and interquartile ranges. The frequency distributions of categorical variables were compared using a chi-square (X^2^) test. Based on diagnosis domains, the Kruskal-Wallis test compared biochemical, dietary, and anthropometric variables across different groups. A logistic regression analysis determined the association between these parameters and nutritional diagnoses in the different domains. Variables were included in the multivariate model using the “Intro” method, which involves including all predictor variables in the model simultaneously. This method allows for the evaluation of the contribution of each predictor while controlling for the others. “Intro” is commonly used when the research focuses on variables selected *a priori*. Variables with a significant *p*-value ≤0.05 or those with biological plausibility remained in the final model.

For all tests, a *p*-value ≤0.05 was considered statistically significant. The analyses were performed using the IBM Statistical Package for Social Sciences (IBM SPSS), version 21.0 (SPSS Inc., Chicago, IL, United States).

## Results

Data from 2,050 patients were analyzed, 55.3% were women. The median age was 57 years for women and 54 years for men. Nutritional diagnoses were categorized by domain, with the most prevalent ingestion domain representing 55.9% of the sample (1,146 individuals). The most common diagnosis within this domain was excessive carbohydrate intake, affecting 23.5% of patients (482 individuals). In the behavioral domain, comprising 32.7% of the sample (671 individuals), the most frequent diagnosis was undesirable food choices, which were present in 20.2% (415 individuals). Finally, in the clinical domain, which accounted for 10.2% of the sample (208 individuals), the predominant diagnosis was overweight or obesity, with a prevalence of 10.0% (205 individuals). In 1.2% of the participants, no nutrition problem was identified; therefore, no nutrition diagnosis was established during the visit (Table 1).

**Table 1:** Diagnoses by domain.

Domain	n/%	Most prevalent diagnosis within each domain	n/%
Ingestion	1,146 (55.9%)	Excessive Carbohydrate Intake	482 (23.5%)
Behavioral	671 (32.7%)	Undesirable Food Choices	415 (20.2%)
Clinical	208 (10.2%)	Overweight or Obesity	205 (10.0%)
Without diagnosis	25 (1.2%)	No diagnosis	25 (1.2%)

Regarding anthropometric variables, individuals in the Ingestion domain had a median weight of 74.5 kg (65.0–86.0 kg), while those in the Clinical domain had 80.6 kg (71.2–92.0 kg), and the Behavioral domain had 76.1 kg (66.0–86.8 kg). Participants without a diagnosis had a median weight of 74.5 kg (65.0–86.0 kg), with significant differences between groups (*p* < 0.001). Similarly, individuals diagnosed in the Ingestion domain had a median BMI of 28.6 kg/m^2^ (25.6–32.0 kg/m^2^), while those in the Clinical domain had 30.6 kg/m^2^ (28.0–34.6 kg/m^2^) and in the Behavioral domain had 28.8 kg/m^2^ (25.9–32.2 kg/m^2^). Participants without a diagnosis had a median BMI of 26.5 kg/m^2^ (24.9–29.0 kg/m^2^), with significant differences between groups (*p* < 0.001). For waist circumference, males in the Ingestion domain had a median measurement of 99.4 cm (92.5–107.3 cm), while those in the Clinical domain had a median of 103.7 cm (98.7–115.0 cm). Males in the Behavioral domain showed a median of 98.7 cm (92.5–107.1 cm), and those without a diagnosis had a median of 97.9 cm (88.6–99.3 cm), with significant differences between groups (*p* < 0.001). For females, the median measurements were 96.0 cm (88.0–103.0 cm) for the Ingestion domain, 98.7 cm (92.0–108.3 cm) for the Clinical domain, 97.0 cm (89.0–104.5 cm) for the Behavioral domain, and 95.0 cm (88.1–101.0 cm) for those without a diagnosis, again with significant differences (*p* = 0.026). As for body fat percentage, males in the Ingestion domain had a median of 31.4% (27.8–34.5%), while those in the Clinical domain had 33.6% (30.2–36.2%). Males in the Behavioral domain showed a median of 31.1% (28.1–34.7%), and those without a diagnosis had 30.3% (27.1–31.7%), with significant differences (*p* = 0.003). For females, the median body fat percentages were 38.2% (35.1–41.4%) in the Ingestion domain, 39.8% (36.8–42.9%) in the Clinical domain, 38.5% (35.1–42.2%) in the Behavioral domain, and 36.1% (33.2–40.3%) for those without a diagnosis, all showing significant differences (*p* < 0.001; Table 2).

**Table 2:** Anthropometric variables by nutritional diagnosis.

Variable	Ingestion	Clinical	Behavioral	Without diagnosis	Intergroup differences *p*
Weight (kg)	74.5 (65.0–86.0)*	80.6 (71.2–92.0)*	76.1 (66.0–86.8)*	74.5 (65.0–86.0)	<0.001
BMI (kg/m^2^)	28.6 (25.6–32.0)*	30.6 (28.0–34.6)*	28.8 (25.9–32.2)	26.5 (24.9–29.0)*	<0.001
Waist Circumference (cm) Male	99.4 (92.5–107.3)*	103.7 (98.7–115.0)*	98.7 (92.5–107.09)	97.9 (88.6–99.3)*	<0.001
Waist Circumference (cm) Female	96.0 (88.0–103.0)**	98.7 (92.0–108.3)**	97.0 (89.0–104.5)	95.0 (88.1–101.0)**	0.026
Body Fat Percentage - Male	31.4 (27.8–34.5)**	33.6 (30.2–36.2)**	31.1 (28.1–34.7)**	30.3 (27.1–31.7)**	0.003
Body Fat Percentage - Female	38.2 (35.1–41.4)	39.8 (36.8–42.9)*	38.5 (35.1–42.2)	36.1 (33.2–40.3)*	<0.001

Kruskal-Wallis Test * < 0.001, ** < 0.05.

The barriers to adherence to a nutritional plan in patients with T2D are outlined in Table 3. The most commonly reported barrier was a lack of information about a correct diet, affecting 25.7% of the sample (527 individuals), distributed as follows: 7 with no diagnosis, 294 in the Ingestion domain, 178 in the Behavioral domain, and 48 in the Clinical domain. The second most common barrier was frequently eating away from home, affecting 23.7% of the sample (485 individuals), with 6 having no diagnosis, 265 in the Ingestion domain, 169 in the Behavioral domain, and 45 in the Clinical domain. Additionally, 401 individuals (19.6%) reported no barriers, 304 (14.8%) cited a lack of time to prepare meals, 221 (10.8%) reported denial or refusal to make dietary changes, 93 (4.5%) mentioned economic problems, and 17 (0.8%) noted a lack of understanding of instructions.

**Table 3:** Barriers to adherence to a nutritional plan in T2D according to the diagnosis domain.

	Without diagnosis	Ingestion domain	Behavioral domain	Clinical domain	n
Lack of information about the correct diet	7	294	178	48	527 (25.7%)
I eat away from home most of the time	6	265	169	45	485 (23.7%)
None	7	221	127	46	401 (19.6%)
Lack of time to prepare my meals	4	162	99	39	304 (14.8%)
Denial or refusal to make changes in my diet	1	139	64	17	221 (10.8%)
Economic problems	0	57	26	10	93 (4.5%)
I did not understand the instructions	0	7	7	3	17 (0.8%)

Dietary variables related to nutritional diagnoses are summarized in Table 4. The daily caloric intake showed significant differences between groups (*p* < 0.001). The Ingestion domain group had a median intake of 1568.5 calories (1279.7–1907.5), the Clinical domain group had 1403.0 calories (1212.7–1718.7), the Behavioral domain group consumed 1443.0 calories (1221.0–1653.0), while individuals without a diagnosis had a median intake of 1377.0 calories (1300.5–1514.5). Carbohydrate intake also varied significantly across groups (*p* < 0.001). The ingestion domain group had a median carbohydrate percentage of 46.9% (39.0–52.0), compared to 43.0% (38.0–46.0) in the Clinical group, 43.0% (38.0–47.0) in the behavioral group and 41.0% (38.5–45.0) in those without a diagnosis. Protein intake was significantly different as well (*p* = 0.002). In the Ingestion domain, the median protein percentage was 19.0% (17.0–22.0), slightly lower than the Clinical domain at 19.5% (18.0–22.0) and the Behavioral domain at 20.0% (18.0–22.0). Individuals without a diagnosis had a median protein percentage of 21.0% (19.5–22.5). Lastly, fat percentage differed significantly between groups (*p* < 0.001). The Ingestion domain had a median fat percentage of 34.0% (29.0–41.0), the Clinical domain had 38.0% (35.0–41.7), the Behavioral domain had 37.0% (33.0–41.0), and those without a diagnosis had 38.0% (35.0–40.5).

**Table 4:** Dietary variables by nutritional diagnosis.

Variable	Ingestion	Clinical	Behavioral	Without diagnosis	Intergroup differences *p*
Calories/ day	*1568.5 (1279.7–1907.5)	*1403.0 (1212.7–1718.7)	*1443.0 (1221.0–1653.0)	1,377 (1300.5–1514.5)	<0.001
Carbohydrates (%)	**46.9 (39.0–52.0)	**43.0 (38.0–46.0)	**43.0 (38.0–47.0)	**41.0 (38.5–45.0)	<0.001
Protein (%)	***19.0 (17.0–22.0)	19.5 (18.0–22.0)	***20.0 (18.0–22.0)	21 (19.5–22.5)	0.002
Fat (%)	****34.0 (29.0–41.0)	****38.0 (35.0–41.7)	****37.0 (33.0–41.0)	38 (35.0–40.5)	<0.001

Prueba Kruskal-Wallis *< 0.001, **< 0.001, ***0.001, ****<0.001.

The metabolic variables according to nutritional diagnosis are outlined in Table 5. HbA1c levels varied significantly among the groups, with the Ingestion domain group showing a mean HbA1c of 7.7% (6.4–10.2), the Clinical domain at 6.6% (6.1–8.5), the Behavioral domain at 7.9% (6.5–10.4), and those without a diagnosis at 6.2% (5.7–8.1; *p* < 0.001). Similarly, glucose levels differed across domains, with median values of 130.0 mg/dL (104.0–188.0) for the Ingestion group, 117.0 mg/dL (101.0–148.0) for the Clinical group, 132.0 mg/dL (104.0–181.0) for the Behavioral group, and 108.0 mg/dL (90.0–146.5) for those without a diagnosis (*p* < 0.001). LDL cholesterol did not show significant intergroup differences (*p* = 0.165). The median levels were 115.0 mg/dL (92–137.0) for both the Ingestion and Behavioral domains, 108.0 mg/dL (90.0–129.7) for the Clinical domain, and 88.0 mg/dL (72.0–124.5) for those without a diagnosis. Triglyceride levels were also not significantly different (*p* = 0.54), with values of 170.0 mg/dL (122.0–242.0) for the Ingestion group, 163.5 mg/dL (118.2–225.5) for the Clinical group, 167.0 mg/dL (125.0–230.0) for the Behavioral group, and 113.0 mg/dL (96.5–158.0) for those without a diagnosis.

**Table 5:** Metabolic variables by nutritional diagnosis.

Variable	Ingestion domain	Clinical domain	Behavioral domain	Without diagnosis	Intergroup differences *p*
HbA1c (%)	⭑*7.7 (6.4–10.2)	*6.6 (6.1–8.5)	⭑*7.9 (6.5–10.4)	⭑6.2 (5.7–8.1)	<0.001
Glucose (mg/dl)	**130.0 (104.0–188.0)	**117.0 (101.0–148.0)	**132.0 (104.0–181.0)	108 (90.0–146.5)	<0.001
LDL Cholesterol (mg/dl)	115 (92–137.0)	108 (90.0–129.7)	115 (92.0–137.0)	88 (72.0–124.5)	0.165
Triglycerides (mg/dl)	170 (122.0–242.0)	163.5 (118.2–225.5)	167 (125.0–230.0)	113 (96.5–158.0)	0.540
ACR (g/mg)	***9.5 (5.7–22.1)	***8.1 (4.6–16.1)	8.4 (5.0–19.0)	6.3 (3.9–17.1)	0.007

Prueba Kruskal-Wallis *< 0.001, **0.001, ***0.025, ⭑0.05.

However, the albumin-to-creatinine ratio (ACR) did show significant intergroup differences (*p* = 0.007). The Ingestion group had a median ACR of 9.5 g/mg (5.7–22.1), the Clinical group 8.1 g/mg (4.6–16.1), the Behavioral group 8.4 g/mg (5.0–19.0), and those without a diagnosis 6.3 g/mg (3.9–17.1). The Kruskal-Wallis test confirmed significant differences among groups for HbA1c (*p* < 0.001), glucose (*p* < 0.001), and ACR (*p* = 0.025).

The analysis of 2,050 patients categorized nutritional diagnoses into three domains: Ingestion (55.9%), Behavioral (32.7%), and Clinical (10.2%), with excessive carbohydrate intake, undesirable food choices, and overweight/obesity being the most prevalent issues in each domain, respectively. The main barriers to dietary adherence included lack of information and eating away from home. Caloric intake and macronutrient distribution also varied across groups, with higher carbohydrate consumption observed in the Ingestion domain. Significant differences were noted in metabolic variables such as HbA1c and glucose, with the highest levels found in the Behavioral and Ingestion domains, respectively.

## Discussion

The Nutritional Care Process (NCP) is a tool designed to enhance patient care by using standardized language, enabling clear communication among nutrition professionals and healthcare teams (14). Its four steps—assessment, diagnosis, intervention, and monitoring—provide a structured approach to identifying nutritional problems, developing interventions, and tracking progress. Standardized language, such as the Nutrition Care Process Terminology (NCPT), is essential for ensuring consistency and effectiveness in documenting nutritional interventions (13).

In diabetes care, Medical Nutrition Therapy (MNT) is a cornerstone and should be implemented by trained nutrition professionals as part of a diabetes self-care education program (15). The NCP was adopted by the CAIPaDi program to streamline patient-centered nutritional assessments, prioritize diagnoses, and tailor interventions. Despite its benefits, studies on NCP application and its effect on outcomes for patients with T2D remain limited (16, 17). Although the International Confederation of Dietetic Associations and the Academy of Dietetics and Nutrition support the NCP’s use (8, 13), its adoption in outpatient settings has been slower, partly due to challenges such as time constraints and the lack of an electronic infrastructure to facilitate documentation (8, 14–20). The implementation of the NCP and NCPT in outpatient settings, such as diabetes care, faces barriers like difficulties with terminology, integration into daily practice, and varying adoption levels across practice areas. Electronic systems with automated templates can address these challenges by streamlining documentation and improving care quality for T2D patients (3).

Our study aligns with the work of Colin et al. (16), one of the few that assess nutritional diagnoses using NCP terminology in people with diabetes. Interestingly, our findings show a higher prevalence in the Ingestion domain (over 50% of cases), in contrast to Colin et al.’s emphasis on behavioral-environmental issues. This discrepancy highlights the need to explore how nutritional diagnoses vary across populations and settings. Additionally, this variability emphasizes the importance of segmenting the type 2 diabetes population. A recent study on diabetes subgroups identified different patient classes based on age, comorbidities, and disease duration, linking these subgroups to healthcare use and complication risks. Analyzing these subgroups alongside their nutritional diagnoses could offer a clearer perspective on nutritional interventions and outcomes (20). Furthermore, a study in Singapore highlighted how population segmentation in T2D patients can effectively connect specific segments to healthcare utilization and risks, providing valuable insights for tailored interventions (21).

The limited data on prevalent nutritional diagnoses and their direct or indirect associations with metabolic, anthropometric, and dietary control parameters highlights the significance of our study (17). By understanding these nutritional diagnoses, healthcare professionals can tailor interventions more effectively, ultimately improving metabolic control and the quality of life in patients with T2D (15).

Moving forward, exploring the implications of nutritional diagnoses for patients with T2D is critical. Identifying common nutritional issues can support the development of targeted, personalized interventions. Additionally, population segmentation by diagnosis could allow for evaluating differential risks of complications and healthcare utilization (17). Future research should focus on patient transition between diagnostic groups over time and what this means for long-term disease management (22).

Our study found that individuals with higher weight, BMI, and waist circumference were more likely to have clinical nutritional issues. At the same time, poor glycemic control was often linked to behavioral and environmental problems. Additionally, those with higher energy intake and a greater percentage of carbohydrates in their diet were primarily affected by ingestion-related issues. This knowledge is vital in focusing the initial nutritional assessment to inform subsequent interventions, ultimately leading to better health outcomes (5, 16).

While the NCP’s use in clinical nutrition has been proven effective, its implementation outside hospital settings, particularly for patients with diabetes, remains understudied. Tailoring nutritional interventions has shown significant impacts on metabolic control in T2D patients, yet research on NCP and its impact on long-term metabolic outcomes in diabetes care is scarce (20). Further research is needed to assess the long-term impact of interventions guided by NCP on metabolic control, health complications, and healthcare use in people with diabetes (8, 23).

Limitations of our study include the need for comprehensive information on whether patients maintain the same nutritional diagnosis throughout their lifetime or transition between diagnostic groups (20). Additionally, we did not evaluate the implications of nutritional diagnoses on patients with complications, an area for future exploration. Considering that population segmentation in patients with T2D can help assess clinical outcomes (24), incorporating such segmentation could provide insights into how nutritional diagnoses affect patients’s risk complications and healthcare utilization (21).

This study contributes to understanding the prevalence and implications of nutritional diagnoses in T2D. It suggests that adopting NCP and NCPT in outpatient care can enhance personalized nutrition interventions. However the results are limited to the population studied in the CAIPaDi, highlighting the need for studies to optimize their broader application.

## Conclusion

The nutritional assessment of people living with type 2 diabetes revealed that the predominant diagnoses were related to the domains of ingestion, followed by behavioral/environmental factors. Ingestion-related nutritional diagnoses were associated with hypertriglyceridemia and a higher intake of energy from carbohydrates. Conversely, behavioral/environmental diagnoses were associated with higher HbA1c levels, suggesting challenges in optimal disease management.

At the initial assessment, participants without a clear nutritional diagnosis showed better metabolic control, lower BMI, waist circumference, weight, and balanced macronutrient intake. These results highlight that poor metabolic control in diabetes is mainly tied to behavioral and environmental issues. Additionally, factors related to increased body weight are predominantly within the clinical domain, while those connected to excessive energy and carbohydrate intake belong to the ingestion domain.

This information can guide the development of tailored nutritional therapies to optimize disease management and promote a healthier lifestyle. We recommend implementing personalized nutritional interventions within an interdisciplinary framework that involves various specialties. For future research, we suggest conducting longitudinal studies to examine the relationship between nutritional diagnoses and metabolic control in patients with type 2 diabetes, as well as assessing the impact of interventions based on this model.

## Data Availability

The raw data supporting the conclusions of this article will be made available by the authors upon on reasonable request.

## Members of the group of study CAIPaDi

María Teresa Alcántara-Garcés, Denise Liliana Arcila-Martínez, Rodrigo Arizmendi-Rodríguez, Humberto Del Valle-Ramírez, Arturo Flores García, Mariana Granados-Arcos, Héctor Manuel Infanzón-Talango, Arely Hernández Jasso, Diana Hernández Juárez, Claudia Lechuga-Fonseca, Angélica Palacios-Vargas, Liliana Pérez-Peralta, Maureen Alexis Acosta Mireles, David Rivera de la Parra, Alejandra Estefanía Montserrat Rodríguez-Ramírez, Francis Evelyn Rojas-Torres, Sandra Sainos-Muñoz, José Luis Cárdenas Fragoso, Héctor Rafael Velázquez-Jurado, Andrea Liliana Villegas-Narvaez, Luz Elena Urbina-Arronte, Carlos A. Aguilar-Salinas, Francisco J Gómez-Pérez, David Kershenobich-Stalnikowitz.
